# How equitable is bed net ownership and utilisation in Tanzania? A practical application of the principles of horizontal and vertical equity

**DOI:** 10.1186/1475-2875-8-109

**Published:** 2009-05-21

**Authors:** Fred Matovu, Catherine Goodman, Virginia Wiseman, William Mwengee

**Affiliations:** 1 Faculty of Economics and Management, Makerere University, P.O Box, 7062 Kampala, Uganda; 2 Health Policy Unit, London School of Hygiene & Tropical Medicine, London WC1E 7HT, UK; 3 KEMRI/Wellcome Trust Research Programme, PO Box 43640, Nairobi, Kenya; 4 Gates Malaria Partnership, London School of Hygiene and Tropical Medicine, 50 Bedford Square, London, UK; 5 WHO, Country Office, Dar es Salaam, Tanzania

## Abstract

**Background:**

Studies show that the burden of malaria remains huge particularly in low-income settings. Although effective malaria control measures such as insecticide-treated nets (ITNs) have been promoted, relatively little is known about their equity dimension. Understanding variations in their use in low-income settings is important for scaling up malaria control programmes particularly ITNs. The objective of this paper is to measure the extent and causes of inequalities in the ownership and utilisation of bed nets across socioeconomic groups (SEGs) and age groups in Tanga District, north-eastern Tanzania.

**Methods:**

A questionnaire was administered to heads of 1,603 households from rural and urban areas. Households were categorized into SEGs using both an asset-based wealth index and education level of the household head. Concentration indices and regression-based measures of inequality were computed to analyse both vertical and horizontal inequalities in ownership and utilisation of bed nets. Focus Group Discussions (FGDs) were used to explore community perspectives on the causes of inequalities.

**Results:**

Use of ITNs remained appallingly low compared to the RBM target of 80% coverage. Inequalities in ownership of ITNs and all nets combined were significantly pro-rich and were much more pronounced in rural areas. FGDs revealed that lack of money was the key factor for not using ITNs followed by negative perceptions about the effect of insecticides on the health of users. Household SES, living within the urban areas and being under-five were positively associated with bed net ownership and/or utilisation.

**Conclusion:**

The results highlight the need for mass distribution of ITN; a community-wide programme to treat all untreated nets and to promote the use of Long-Lasting Insecticidal nets (LLINs) or longer-lasting treatment of nets. The rural population and under-fives should be targeted through highly subsidised schemes and mass distribution of free nets. Public campaigns are also needed to encourage people to use treated nets and mitigate negative perceptions about insecticides.

## Background

Much effort is currently being directed towards stimulating the demand for Insecticide Treated Nets (ITNs) in African communities. ITNs have been shown to reduce child mortality by about 20%, saving six lives for every 1000 children under five years of age protected per year in sub-Saharan Africa (SSA) [1]. The cost-effectiveness of ITNs has also been demonstrated [2,3]. Despite this, current rates of net coverage remain disappointingly low in most African countries.

While a wide literature exists on ITN efficacy [4,5], there is an urgent need for more information on the equity of bed net ownership and utilisation to inform the scaling up of malaria control programmes. Inequities can be considered from both a horizontal and vertical perspective [6]. Horizontal equity refers to a situation whereby individuals or social groups with the same level of need are treated equally. Vertical equity relates to cases where people with unequal needs are treated proportionately differently. For example, in the case of ITNs, the under-fives could be considered to be in greater need due to their low immunity to malaria. This study therefore analyses the nature and extent of inequalities in ownership and use of bed nets across socioeconomic groups (SEG) (from a horizontal equity perspective), and age (from a vertical equity perspective).

The relatively small number of studies available suggests that wealth is a key determinant of the demand for bed nets. A study from The Gambia found that wealthier households owned significantly more nets than their poorer counterparts [7]. Data from an ITN social marketing project in Tanzania demonstrated that the least poor quartile of the population were 2.74 times more likely to own a bed net than the poorest quartile [8]. There is also some evidence that educational attainment is associated with malaria-specific knowledge and uptake of preventive measures more generally [9,10]. Other factors found to influence net use include location of the household and socio-demographic factors, such as gender, marital status, occupation, family size, etc [7,9,10].

This scenario of low uptake by the poor is by no means a phenomenon unique to bed nets. Similar disparities have been found in studies investigating access and use of malaria treatment [11,12]. Studies focusing on uptake of other types of health care services show significant pro-rich inequalities across SEGs [13-16]. This is despite the fact that many of these services are targeted at the poor.

For malaria to no longer be a major cause of mortality or barrier to economic development, strategies for targeting the poor must be at the forefront of malaria control programmes and donor activities. Such strategies must be underpinned by accurate information about the nature and the causes of inequalities in utilisation. The aims of this study are three-fold. Firstly, to measure the extent of horizontal and vertical inequalities in ownership and use of bed nets across SEGs and age groups. Secondly, to examine the relationship between bed net ownership, utilisation, socioeconomic status and other key determinants. Thirdly, to explore factors undermining bed net uptake. These objectives are addressed in the context of Tanga district of north-eastern Tanzania, a typical low income, malaria endemic African setting.

## Methods

### Study sites

Data were drawn from a household survey (HHS) and focus group discussions (FGD) conducted in rural and urban areas of Tanga district. The climate is monsoonal with short rains from October-December and long rains from March-May, giving an average annual rainfall of about 1000 mm. There are four dominant ethnic groups: the Digo, Bondei, Wazigua and Sambaa, with over 20 other smaller ethnic groups. Tanga district is relatively typical of the Tanzanian mainland in terms of poverty levels, average household size and treatment seeking behaviour [17]. The main economic activities in rural areas are subsistence farming and fishing, with limited cash-cropping. The majority of households in the urban centres are engaged in trade, varying from wholesale stores to petty trade in small markets and along streets. A few others work on sisal plantations.

There were two distribution channels for nets at the time of the HHS: the private sector and non-governmental organizations (NGOs). Drug stores and pharmacies frequently stocked bed nets and other malaria prevention products such as repellents and sprays. Bed nets were also available in some general retail shops. Bed nets were usually packed together with insecticide. At the time of the HHS, the average price for a net was about 3,000/= (about $3), and the insecticide (solution or tablet) cost about 200 shillings ($0.20). Charitable organizations such as Tanga Rotary Club occasionally distributed nets for free but only in small quantities. Population Services International (PSI) was also involved in net promotion through public health campaigns, promoting the net manufacturers of four leading brands, and encouraging bundling nets together with insecticide (Jane Miller, personal communication). A nationwide discounted voucher scheme for ITNs for pregnant women was launched in Tanzania in 2004, but discounted nets only became available in Tanga around mid 2005 after the HHS had been completed.

### Sample

For the HHS a sample of 1,603 households (863 in rural and 740 in urban areas) was selected. The sampling unit was a sub-village in the rural areas and a street in the urban areas. Simple random sampling was used at three stages. First, eight wards were randomly selected in the urban areas and five in the rural areas, representing 80% of the wards in each location. A ward is an administratively demarcated area below the district level, which may comprise three to five villages (rural) or 6–14 streets (urban), and a population of between 2,500 to 26,000 people (about 600 to 5,500 households) [17]. For each of the urban wards sampled, four streets were randomly selected. From the five rural wards, eight villages were randomly selected, and five sub-villages were randomly selected from each village. In total, 32 streets (22% of all streets) and 41 sub-villages (32% of all sub-villages) were selected. A sub-village had between 20 and 50 households, and a street between 100 and 150 households. For logistical reasons, convenience sampling was used to select households from within sub-villages and streets. The number of households interviewed ranged between 5–30 per sub-village and 20–25 per street.

In addition, sixteen FGDs (eight for women and eight for male household heads) involving between eight and 14 people each were conducted within four villages in the rural wards and four streets in the urban wards. The villages and streets were of two categories: those which were relatively poor (two rural, two urban) and those which were relatively better off (two rural, two urban) based on the average wealth-index scores from the household survey data and the investigators' knowledge of the study areas. Different FGDs were held for the poorest as they may not feel comfortable and powerful enough to express their views among those of a relatively higher social status. Individual participants were purposively selected with the help of community leaders to reflect the views of different age, religious and ethnic groups.

### Data collection

Structured HHS interviews were conducted with household heads or their spouse. Interviews were staggered over an 18-month period (September 2003 to February 2005) to capture any seasonal variations in reported fever and household activity. The survey provided data on SES in terms of household head education and asset possession. Data were collected on the family members sleeping under a net the night before the survey, the number of nets per household, and expenditure on net purchase, re-treatment and repair. A treated net (ITN) was one that had been treated with insecticide within the past six months. Data were also collected on use of other malaria prevention products in the past two weeks (mosquito repellents, coils, aerosols and indoor sprays, using smoke, clearing vegetation and cleaning the environment around homesteads). The FGDs explored community perspectives on barriers to the use of ITNs, and were conducted between November and December, 2006. All data collection was conducted in Swahili.

The study received ethical approval from the National Institute of Medical Research (NIMR) of the Republic of Tanzania, and from London School of Hygiene and Tropical Medicine (LSHTM). Clearance was also obtained from the Regional Medical Office, Tanga, and from Bombo hospital where the study was based. Verbal consent to participate was sought from all categories of participants.

### Analysis

HHS data were double entered in Microsoft Access and analysis was performed in STATA 8, adjusting for stratification (rural/urban) and clustering within streets and sub-villages using survey commands (e.g. svyset, [pw; strata; psu]). For pooled urban and rural analysis sampling weights were computed to adjust for the different probability of being selected in the urban and rural areas [18]. The FGDs were tape-recorded, transcribed and translated into English by a fieldworker, and subjected to manual content analysis.

To analyse horizontal inequities in the ownership of bed nets at household level, the proportion of households owning at least one bed net within the rural areas and the urban areas and across SEGs was computed. In the absence of data on local malaria transmission, all households were assumed to be in the same need of protection. Under-fives were considered to be in greater need of nets than over-fives due to their low malaria immunity. Vertical inequities were analysed by considering the proportion of under-fives sleeping under a net compared to other age groups. Concentration indices (CI), equity ratios and a logit regression model [19,20] were computed to analyse inequalities in ownership of treated and all nets combined (treated and untreated) across SEGs both in rural and urban areas. The concentration index ranges between -1 to + 1 with a positive index indicating pro-rich inequalities [18]. An equity ratio is a simple measure of inequity between the poorest and least poor SEGs (e.g. rich/poor). A ratio greater than one imply pro-rich inequities. Pearson chi-square and t-test statistics were used to test for statistical significance at the 5% level.

The study employed two measures of SES: education of household head and an asset-based wealth index, both applied at the household level. The HHS collected data on education level of the household head in terms of years of formal schooling completed. Formal schooling excluded the years spent in *koranic *(religious) schools. Five educational classes were constructed based on national examinations in Tanzania: (1) no education; (2) lower primary (1–4 years); (3) upper primary (5–7 years); (4) secondary (8–11 years) and; (5) post-secondary (12+ years). A wealth index was computed for each household based on 14 household assets using principal components analysis (PCA) [21]. The assets were radio, bicycle, television, iron bed, tin/iron roof, motorbike, watch, sponge mattress, cattle, sheep, goats, chicken, electric cooking and purchased wood fuel. The terms 'iron-roof' and 'tin-roof' are used interchangeably to refer to houses roofed with corrugated iron sheets, tin and other manufactured metal. These items were considered to highlight the relative income position of the household, and therefore the respective ability to afford malaria preventive measures. The asset index was calculated over the pooled data for rural and urban areas, and households were categorized into quintiles based on their PCA score.

## Results

### HHS sample characteristics

Basic socio-demographic characteristics of the household head are presented in Table 1. There were statistically significant differences between rural and urban areas for all categories, with for example household heads in urban areas more likely to be male and to have more years of education.

**Table 1:** Socio-demographic characteristics of household head

Indicator	Rural (%)	Urban (%)	Overall (%)	Chi-square/t stat*	p-value
Male-headed households	676 (78.5)	636 (85.9)	1312 (81.9)	15.3	0.0002
Female-headed households	185 (21.5)	104 (14.1)	289 (18.1)		

Ethnicity					
Wazigua	40 (4.6)	74 (10.0)	114 (7.1)		
Sambaa	57 (6.6)	145 (20.0)	202 (12.6)	207.9	<0.0001
Bondei	24 (2.8)	65 (8.8)	89 (5.6)		
Digo	431 (50.1)	146 (19.8)	577 (36.0)		
Other	309 (35.9)	310 (41.9)	619 (38.7)		
**Total**	**861 (100)**	**740 (100)**	**1601 (100)**		

Marital Status					
Married	643 (74.5)	626 (84.6)	1269 (79.3)		
Unmarried	42 (4.9)	27 (3.6)	69 (4.3)	26.08	<0.0001
Divorced	95 (11.0)	48 (6.5)	143 (8.9)		
Widowed	83 (9.6)	39 (5.3)	122 (7.6)		
**Total**	**863 (100)**	**740 (100)**	**1603 (100)**		

Average years of schooling	5.4	8.4	6.8	19.16	<0.0001
Average age	44.7	42.5	43.1	-3.1	0.002
Average family size	5.2	5.7	5.5	-3.85	<0.001

Education level					
None	148 (17.2)	20 (2.7)	168 (10.5)		
Lower primary	136 (15.8)	54 (7.3)	189 (11.8)	341.62	(<0.0001)
Upper primary	524 (60.7)	381 (51.5)	904 (56.5)		
Secondary	43 (5.0)	156 (21.1)	199 (12.4)		
Post-secondary	12 (1.4)	129 (17.4)	141 (8.8)		
**Total**	**863 (100)**	**740 (100)**	**1603 (100)**		

Wealth quintile					
Poorest 1	292 (33.8)	29 (3.9)	321 (20.0)		
2nd quintile	240 (27.8)	81 (11.0)	321 (20.0)	452.70	(<0.0001)
3rd quintile	185 (21.4)	135 (18.2)	320 (20.0)		
4th quintile	121 (14.0)	200 (27.0)	321 (20.0)		
Least Poor 5	25 (2.9)	295 (39.9)	320 (20.0)		
**Total**	**863 (100)**	**740 (100)**	**1603 (100)**		

* The last two columns give estimates of the Pearson's chi-square (or t-statistic for years of school, age and family size) and the p-value, testing for statistical difference between rural and urban areas.

The majority of household heads both in the rural and urban areas had seven years of schooling (upper primary) or less. The proportion of household heads with secondary education and above was higher in the urban areas (38%) compared to just 6% in the rural area. The large proportion of those who completed upper primary (7 years), both in rural and urban areas, reflects the fact that primary education in Tanzania is largely free in government schools. Regarding distribution of households by wealth quintiles, the majority of households in the lower SEG were located in rural areas. Detailed results of the PCA are presented in additional File 1.

The two SES measures (education and asset index) were significantly correlated (Spearman Rank Correlation Coefficient of 0.50; p < 0.001), indicating that they appeared to be measuring a similar underlying phenomenon. However, the correlation was not close to 1, justifying the use of both SES measures in the analysis.

### Ownership and utilisation of bed nets by location and age groups

Ownership of treated nets and all nets combined is shown in Table 2 by a range of household characteristics. Ownership of all nets combined and treated nets was higher in the urban areas than rural areas. For instance, the average number of nets owned by a household was about 2.5 times higher in urban areas. By age group, utilisation was higher for under-fives compared to over-fives both within the rural and urban areas.

**Table 2:** Bed net ownership, utilisation and expenditure by household and individual characteristics

	**Any net**			**Treated net**		
	Rural	Urban	Overall	Rural	Urban	Overall

Ownership						
All households	49.7%	89.7%	79.5%	8.8%	48.0%	38.0%
Gender of household head						
Male	51.6%	90.6%	81.3%	9.6%	50.2%	40.5%
Female	43.0%	84.6%	70.3%	5.9%	34.6%	24.7%

Marital status of household head						
Married	51.8%	90.9%	81.8%	9.8%	50.5%	41.1%
Unmarried	43.6%	83.3%	69.0%	5.9%	34.2%	24.0%

Wealth quintile						
Poorest	32.1%	58.6%	38.9%	3.1%	17.2%	6.7%
2nd quintile	48.3%	85.1%	68.1%	7.0%	44.4%	27.1%
3rd quintile	60.0%	92.6%	83.3%	10.3%	43.7%	34.1%
4th quintile	70.2%	87.5%	84.9%	19.0%	41.5%	38.1%
Least poor	94.2%	92.0%	94.1%	32.0%	58.3%	57.7%

Education level of household head						
None	32.4%	80.0%	47.4%	2.0%	10.0%	4.5%
Lower primary	40.4%	77.8%	61.9%	4.4%	24.1%	15.7%
Upper primary	54.6%	87.9%	78.3%	10.3%	45.7%	35.5%
Secondary	72.1%	96.1%	94.4%	25.6%	54.5%	52.3%
Post-secondary	75.0%	93.8%	93.3%	16.7%	62.8%	61.6%
Average number of nets owned per household	0.9	2.6	2.2	-	-	-

Utilisation						
Age of individuals						
Under-fives	46.0%	86.0%	74.0%	10.0%	47.0%	36.0%
Over-fives	31.0%	79.0%	68.0%	7.0%	43.0%	35.0%
Expenditure						
Per capita expenditure on nets over previous six months (US$)*	0.58	1.74	1.45	-	-	-

*Average exchange rate at time of data collection was US$1 = 1000 Tanzanian Shillings.

### Horizontal equity

Socioeconomic inequalities in the ownership of bed nets across SEGs are shown in Table 3. Equity ratios (least poor/poorest SEG) and concentration indices are presented for net ownership (ITNs and all nets combined) at the household level. The equity ratios show that the proportion of households owning at least one net in the least poor SEG was between two and three times that of the poorest SEG in rural areas, and 1.2 to 1.6 times in urban areas, depending on the SES measure used. Inequities in ownership of treated nets were greater. The ownership of treated nets by the least poor varied between nine and 11 times that for the poorest SEGs in rural areas and between three and six times in the urban areas based on education and the wealth index respectively. Concentration indices showed that inequities in ownership of ITNs were significantly pro-rich within the rural areas and overall both by education and wealth quintiles but were not significant in the urban areas. A similar trend emerged for all nets combined except that inequalities within the urban areas based on education were now significantly pro-rich.

**Table 3:** Socioeconomic inequalities in bed net ownership

Intervention	Inequality measure	Rural	Urban	Overall
Ownership of ITNs	Equity ratio by:			
	Wealth	10.7	3.4	8.3
	Education class	8.5	6.3	12.4
	C.I (t-statistic) by:			
	Wealth	0.368 (3.67)*	0.093 (1.95)	0.276 (2.60)*
	Education class	0.276 (2.93)*	0.117(1.70)	0.234 (2.01)*

Ownership of all nets	Equity ratio by:			
	Wealth	2.9	1.6	2.4
	Education class	2.3	1.2	2
	C.I (t-statistic) by:			
	Wealth	0.169 (3.98)*	0.027 (0.96)	0.138 (2.30)*
	Education class	0.108 (2.21)*	0.028 (2.36)*	0.089 (2.07)*

* Concentration indices significant at 5% level; t-statistic are given in brackets

The determinants of owning at least one net within a household were also investigated using multivariate analysis. The definitions of the variables for the logit model are presented in Table 4 and the regression results are given in Table 5 (panel A).

**Table 4:** Definition of variables for logit model of bed net ownership/utilization

Variable	Definition	Mean
Dependent variables		
Any net	1 if at least one net	0.80
Treated net	1 if at least one net treated in previous 6 months	0.38

Independent continuous variables		
Family size	number of people sharing same roof and source of food	5.54
Age of household head	age of household head in years	43.10
Education class	years of education of household head	7.61
Wealth	household asset-based PCA score	0.61

Independent dummy variables		
Male	1 if household head is a male	0.84
Urban	1 if household located in the urban area	0.75
Under-five	1 if individual is below 5 years of age	0.13
Married	1 if household head is married	0.82
Wazigua	1 if household head is Zigua (reference group)	0.09
Sambaa	1 if household head is Sambaa	0.16
Digo	1 if household head is Digo	0.27
Bondei	1 if household head is Bondei	0.07
Other	1 if households head is of other ethnic group	0.40
Using other prevention measures	1 if household used any other prevention measure over the previous 2 weeks	0.27
Poor road	1 if road to the community is impassable sometime during the year	0.07
Market centre	1 if there is a shop or market in the community	0.98

**Table 5:** Logit regression estimates for the probability of owning a bed net at household level and using a bed net at individual level

	**Panel A: Horizontal equity analysis: Household level** (N = 1603; F < 0.0001)				**Panel B: Vertical equity analysis: Individual level** (N = 8663; F < 0.0001)			
	
Level of Analysis	All Nets		Treated nets		All Nets		Treated nets	
	Marginal/Average effects†		Marginal/Average effects		Marginal/Average effects		Marginal/Average effects	
Explanatory variable	dF/dx	p-value	dF/dx	p-value	dF/dx	p-value	dF/dx	p-value
Family size	0.001	0.973	-0.002	0.656	-0.274	<0.001*	-0.008	<0.001*
Male	-0.032	0.555	-0.01	0.859	-0.056	<0.001*	-0.02	0.023*
Urban	0.228	<0.001*	0.205	<0.001*	0.315	<0.001*	0.192	<0.001*
Married	0.007	0.887	0.043	0.408	0.031	0.051*	0.04	<0.001*
Sambaa	0.072	0.214	0.031	0.496	-0.087	0.003*	0.011	0.546
Digo	-0.085	0.108	-0.102	0.012*	-0.176	0.001*	-0.086	0.001*
Bondei	0.079	0.248	-0.051	0.314	-0.05	0.15	-0.056	0.005*
Other ethnic group	0.006	0.908	-0.078	0.048*	-0.09	0.001*	-0.056	<0.001*
Age of household head	-0.002	0.097	-0.001	0.133	n/a	n/a	n/a	n/a
Under-five	n/a	n/a	n/a	n/a	0.157	<0.001*	0.06	<0.001*
Using other prevention measures	-0.232	<0.001*	-0.006	0.81	-0.182	<0.001*	-0.004	0.684
Education	0.013	0.008*	0.02	<0.001*	0.018	<0.001*	0.02	<0.001*
Wealth	0.11	<0.001*	0.053	<0.001*	0.091	0.001*	0.043	<0.001*
Education-squared	-0.002	0.028*	-0.001	0.073	n/a	n/a	n/a	n/a
Wealth-squared	-0.008	<0.001*	-0.004	<0.001*	-0.007	0.001*	-0.004	<0.001*
Poor Road	0.012	0.698	-0.07	0.085	n/a	n/a	n/a	n/a
Market centre	-0.016	0.764	-0.008	0.906	n/a	n/a	n/a	n/a
Constant	-	-	-	-	-	-	-	-

* Coefficient significant at 5% level

†marginal (average) effects are computed using the *dprobit *command and reflect the effect of a unity change in the continuous (dummy) variable on the dependent variable.

Ownership of at least one net within the household was significantly positively associated with living within an urban area, and household SES (education and wealth), and significantly negatively associated with use of other malaria prevention measures. Marginal and average effects analysis showed that a unit increase in the number of years of education of the household head was associated with an increase in the probability of owning a net of 1.3% points, and of 11% points for a unit increase in the wealth index. The probability of owning a net fell by 23% points if a household used other prevention measures i.e. other prevention products appear to be substitutes for nets. Quadratic terms for education class and wealth were tested. Both were significant and negatively associated with bed net ownership, implying that there is a threshold of SES beyond which an increase in SES leads to smaller and smaller increases in the probability of net ownership. This could reflect households where the risk of malaria is deemed minimal, say due to well protected housing, resulting in less and less demand for bed nets. It could also reflect saturation in net coverage. The results for ownership of a treated bed net were similar, except that using other prevention was no longer significant while being from the Digo and 'other' ethnic sub-populations were significantly negatively associated with owning a treated net.

### Vertical equity

**Figure 1:**
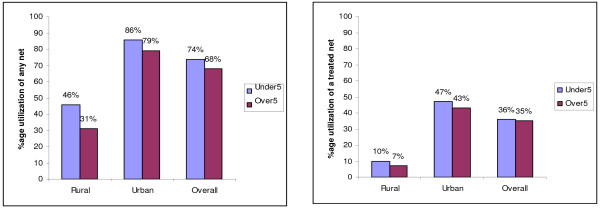
**Utilisation of any net and treated nets by age group**. The nets were categorised into treated nets and all nets combined (treated and untreated). Percentage utilisation of each type of net was then computed for over-fives and under-fives for rural and urban areas and overall. The figure compares the utilisation of each type of net by age group in each location to highlight the vertical equity dimension of bed net use in the study area.

Bed net utilisation by age group was explored by analysis of individual level HHS data for any net and ITNs (Figure 1). The estimates showed that over 50% of rural under-fives were not using any net compared to just 14% within the urban areas. Only 10% of rural under-fives used a treated net compared to 47% within the urban areas. Utilisation of any net among under-fives was significantly greater than for over-fives within the rural villages, urban areas and overall (p < 0.0001 in each case). Utilisation of treated nets by under-fives was significantly greater than for over-fives within the rural areas (p = 0.0095) but not the in urban areas (p = 0.1806) nor overall (p = 0.568).

Utilisation of bed nets across age groups was explored further using a logit model (Table 5 panel B). Being under-five, wealth of household, education level of household head and using other malaria prevention methods were significantly associated with using any net. The probability of using a net increased by 16% points if the individual was below 5 years of age. The probability of using a net increased by 9% points for a unit increase in the wealth index, but fell by 18% points if the household had used any other malaria prevention measure. Other significant predictors positively associated with utilisation of any net were small family size, urban location, being from the Digo ethnic group compared to being a Zigua (the reference ethnic group) and the household head being married and female. Those that were significantly negatively associated with net use were using other malaria prevention measures and the quadratic term for wealth. The results were similar for treated nets except that having a poor access road was now negatively associated with using a treated net and using other prevention measures was no longer significant. Also, the marginal and average effects were correspondingly smaller.

### Community perceptions on barriers to net ownership and utilisation

Lack of financial resources was frequently raised in FGDs, both in rural and urban areas, as a key barrier to obtaining nets. Participants in most FGDs observed that because of low incomes, households tended to give greater priority to very immediate needs such as food, clothing, etc. compared to nets: A woman in a rural village commented:

*'you cannot go to buy a net or ngao (insecticide) when you don't have something to eat, no kerosene etc'*,

and a female participant from a less developed street expressed similar views:

'*if you have 500/= and you want to buy okra and corn, at the same time you need Ngao (insecticide) to treat your net, you have to buy food and let the mosquitoes bite you!*'

Similarly, in some FGDs lack of money was given as the reason for not treating nets even though the insecticide was not considered expensive. If there was no money at hand when nets were washed, people were likely to defer treating the net until the next washing. This reflects the tight budget constraint faced by many households. Participants in most FGDs argued that affordability was a particular challenge in rural areas where households mostly depended on income from low value crops like maize, oranges and coconuts. Borrowing to buy ITNs was not mentioned in any FGDs. Unlike treatment costs, prevention expenditures were not considered as emergency expenses, so the household had to wait until there was money either from farm produce or other household income sources.

Other barriers identified were knowledge and perceptions about nets and net-treatment products; and low perceived risk of infection. Participants in all FGDs demonstrated good knowledge of the link between malaria/fever and mosquitoes. However, participants in some urban and rural FGDs observed that there were still a number of people who were not well-informed about the importance of ITNs. In a discussion with rural women, one participant commented:

'*When you look at us and the clothes we are wearing, would you really think we cannot buy Ngao (insecticide) or a bed net? For me I think we don't have good knowledge about those things and how important they are in fighting malaria*!'

It was pointed out that some people were not using treated nets because the insecticide caused them skin irritation, nose congestion and difficulty in breathing. Others perceived the chemical to be ineffective in repelling and/or killing mosquitoes and therefore a waste of resources. One participant explained that this may have reflected the practice of applying one packet or tablet to 2 or 3 nets – possibly to reduce costs. Some members expressed concerns about the safety of the insecticide, thinking that the chemical could be harmful to their health since it could kill mosquitoes and other insects. In one of the FGDs, a participant commented:

'For me I think Ngao (an insecticide for nets) can damage our lungs since it can even kill mosquitoes!"

Although one could argue that households in urban areas generally have more access to public health information, such 'incorrect' perceptions about ITNs were as frequently mentioned in the urban areas as in the rural areas. In a related discussion, a number of factors affecting susceptibility to mosquitoes and fever/malaria infection were said to influence the use of bed nets. The general view from all FGDs was that children, especially the under-fives, were more prone to mosquito bites and suffered the worst consequences from malaria/fever illness, and that they should be given priority to use a net if one was available. Some adults, especially men, considered themselves to be highly immune to malaria, and therefore were less likely to use a net. A few people mentioned concerns about the small size of available nets and poor supplies in remote areas, but these issues were only raised in a minority of groups, suggesting that supply-side issues were not a major barrier. The general view was that net outlets were widespread including drug shops, pharmacies, general stores, retail shops and vendors in rural markets. Detailed results from the FGDs are reported elsewhere [22].

## Discussion

### Overview of equity in bed net ownership and utilisation

The results revealed consistently pro-rich inequalities in ownership of both treated and all nets, as shown by the equity ratios and concentration indices. After controlling for location and other determinants using regression analysis, SES measured by education class and wealth remained a significantly positive predictor of the ownership and use of ITNs and bed nets in general. This implies that the least poor were more likely to own and use bed nets and ITNs than their poorer counterparts, although all could be considered in equal need of protection. The results therefore do not uphold the principle of horizontal equity (i.e. equal treatment of equals). This was supported by evidence from FGDs, which indicated that lack of money was identified as the most important factor for not using a net, particularly for poorer rural populations.

Overall, ITNs were more unequally distributed across SEGs than all nets combined. This could reflect differences across SEGs in ability to pay the monetary costs of net treatment products, as well as differences in knowledge and perceptions about net treatment. Bivariate analysis based on equity ratios and C.Is showed that inequalities were more pronounced and more likely to be statistically significant in rural areas, perhaps reflecting the higher proportion in the lowest SEGs, for whom affordability would be a particular challenge.

The few studies whose results could be compared directly to our findings also found pro-rich inequalities in net ownership and utilisation [7,23-25]. In The Gambia the household wealth index was significantly associated with the number of nets owned [7,24]. In a study on net use among under-fives in Kenya, children from the least poor households were 10 times more likely to use a net compared to those from the poorest quintile [23]. This is supported by baseline data from intervention studies, which found strong pro-rich inequalities in net use and ownership pre-intervention [5,26-29]. This suggests that inequalities estimated in this study are typical of those found in other low-income settings.

The regression estimates further revealed that family size was negatively associated with net use at the individual level, in contrast to a positive association found in The Gambia [24]. The inverse relationship between family size and net use could be associated with cost whereby on average, larger families would find it costly meeting more basic household needs such as food and are left fewer resources for net purchase.

A household head being married was associated with a higher probability of owning at least one net. Other studies have also shown that unmarried men were less likely to own a net compared with married men [24,30]. -This could be because married men are viewed as economically responsible for their family, or because the wife was more likely to acquire a net during pregnancy or after birth to protect the baby since many parents preferred to provide some form of protection for infants as revealed in the FGDs.

There was a small but significant gender discrimination in net use in favour of females, in contrast to results from the Gambia where no significant gender discrimination was observed [24]. The reasons for the gender differences are less clear. Participants in FGDs believed that both males and females had equal opportunity to use a net. However, discussions regarding perceived risk of infection showed that some men regarded themselves as less prone to malaria.

Finally, net use was significantly associated with ethnicity. The Digo were less likely to use a net compared to the Zigua. However, the FGD participants were less willing to give their views on this result, perhaps to avoid voicing negative sentiments about particular groups, which limited in-depth discussion. McElroy and colleagues also found ethnic differentials in ownership of nets [24]. They suggested that this reflected ethnic differences in tastes or living conditions, which could not be captured by the SES or location variables. Further work on the causes of gender and ethnic differentials in utilisation of malaria control measures is required.

Vertical equity analysis compared net use between under-fives and over-fives. The results indicated that under-fives were more likely to use a net, whether treated or otherwise after controlling for other socio-demographic characteristics, but the differences were relatively small. One could argue based on statistical significance that this indicated some degree of vertical equity, but the small size of the difference between age groups implies that the situation is still likely to be vertically inequitable in public health terms. In absolute terms under-five utilisation of ITNs remained disturbingly low in both rural (10%) and urban (47%) areas, compared to the RBM target of 80% coverage. The FGDs revealed that the under-fives were said to have priority in the use of bed nets and ITNs, but it was unclear why this did not lead to greater preferential utilisation in practice.

### Methodological limitations

The construction of wealth indices always raises methodological challenges [21,31]. Although there is no agreed number of assets to include in PCA, some authors have raised the concern that using relatively few could reduce the power to distinguish the poor from the very poor, possibly leading to an underestimation of inequalities. In addition, the wealth index was computed over pooled data for rural and urban households to allow for direct comparability of households in different areas. While studies on inequality in Mexico [21] and The Gambia [7,24] have used a similar approach, pooling rural and urban data in the PCA process has two potential limitations. Firstly, ownership of assets such as iron-roofed houses tends to vary more in the rural areas than in the urban. This implies that the weight attached to an iron-roofed house would be higher in a rural-specific index, allowing greater discrimination between households. Secondly, assets may have different meanings in rural and urban areas. For example, an electrical appliance is less important if you live in a rural area without electricity, and a bike may be less important if you live in the city where travel distances are shorter. While some studies have confirmed the validity of using an asset index as a SES measure [32], others have found weak correlation between asset indices and household expenditure [31,33]. While both approaches can have weaknesses and there is no clear gold standard, a study in Kenya found wider inequalities in bed net ownership when expenditure was used to proxy SES [31] – indicating that, if anything, inequalities in this analysis may be underestimated.

There are also difficulties in applying the standard definitions of horizontal and vertical equity in empirical analysis. The horizontal equity analysis assumed that all households were in equal need of ITNs. There is some evidence that urban populations have reduced malaria transmission rates compared with their rural counterparts due to pollution of potential breeding grounds and declining open spaces for breeding [34-37]. In addition, the nature of housing units and sleeping patterns greatly influences vulnerability to mosquito bites [10]. However, as no transmission data were available from these sites, the risk was assumed to be equal across urban and rural areas for the purposes of the equity analysis. Conversely, vertical equity analysis assumed that all under-fives were in greater need of protection than over-fives regardless of other household characteristics such as location. To accurately measure need, further information would be required to assess relative vulnerability to malaria across households, which may be affected by sleeping arrangements, housing quality, and local transmission intensity. A further challenge arose in specifying when a distribution is vertically equitable from a public policy viewpoint. The analysis relied on analytical definitions of vertical equity, which consider greater utilisation rates for those proportionately in greater need. However, it is not clear how much higher utilisation rates for under-fives should be compared to over-fives for the situation to be judged vertically equitable. One possibility would be to compare ITN utilisation with malaria prevalence rates across age groups but it is not clear how these two variables should optimally be related to achieve vertical equity, especially as the risk of progression to severe disease also varies by age.

Finally, due to the convenience sampling method used to select households for the survey, it is possible that households at the periphery of villages/streets who may be poorer were less likely to be selected, which could have led to an underestimation of inequalities. However, the authors' knowledge of the study areas and the pre-HHS visits revealed that households within the same sub-village/street were relatively homogenous in terms of their socioeconomic and demographic factors.

## Conclusion

The findings lead to three key policy implications. Firstly, as there are such a high number of untreated nets in use there is need for a community-wide programme to treat all nets, which are not currently treated. Both urban and rural areas should be targeted. If all nets, which are currently untreated, were to be treated, then use of ITNs would significantly improve to levels above 50% both in rural and urban areas. A regular net-treatment campaign at sub-village/street level could be arranged by local leaders and health management teams to treat nets, which have not been treated in the last 9–12 months, and to replace any torn nets. The problems associated with net treatment are being addressed to some degree through the introduction of long-lasting insecticidal nets (LLINs) or longer-lasting treatment of nets, which are currently being promoted in Tanzania. However, LLINs still represent a small proportion of the total nets available, and are more expensive than regular nets bundled with the insecticide. This implies that access to LLINs, particularly for poorer households, is still limited. Secondly, there is the need for a public campaign to encourage people to use ITNs and mitigate the negative perceptions about insecticides used in net treatment.

Thirdly, and most importantly, there is a need to promote greater utilisation of ITNs by reducing the cost of acquiring a treated net. Our data demonstrated striking inequalities across SEGs in ownership and utilisation of treated nets, which were far greater than inequalities for all nets. The poorest were eight to 12 times less likely to own an ITN than the best off quintile depending on the SES measure used. Moreover, FGDs clearly revealed lack of money as the key barrier to using ITNs, both in rural and urban areas. Since our data were collected, Tanzania has begun to address this affordability gap through a programme of vouchers for pregnant women provided at antenatal care, which cover about 75% of the regular market price of an ITN. Vouchers can be exchanged for an untreated net bundled with insecticide at pre-determined retailers (drug shops, pharmacies and retail shops) or government health care facilities upon payment of the remaining balance [38]. It remains unclear to what extent women share the nets with their children after birth.

However, a heated debate continues on the most appropriate approach to achieving equitable and sustainable ITN delivery between those who favour free mass distribution and those who emphasize the advantages of involving and developing the private sector retail market [27,29,39-45]. The proponents of free distribution argue that where it has been used (e.g. Togo, Tanzania, and Zambia) it has achieved higher, faster and equitable coverage of ITNs, and that the distribution of free ITNs can easily be integrated with other free services such as immunization, which already have a well-developed delivery system. They also argue that the possibility of integration reduces the costs to the provider, and increases the chance of reaching those in most need. Furthermore, rapid increases in coverage with free distribution are capable of stimulating long-term donor support. In contrast, those in favour of ITN social marketing involving the private sector question the sustainability of free distribution, given the enormous amount of resources required to reach all those in need. They also argue that free distribution of nets disproportionately benefits those who are relatively richer and do not need subsidies, and that reliance on donors inhibits local capacity building and stifles local empowerment. Moreover, charging for nets could select out those who do not value or need to use ITNs (selection effect) and also induce buyers to use the nets (psychological effect). In addition, the commercial sector is hitherto the main source of nets and the promoters of social marketing believe a strong competitive market leads to higher quality, lower prices and wider availability for all.

Data from several studies indicate that post-intervention inequalities in net use are lower following free distribution during measles vaccination campaigns or stand-alone campaigns than following social marketing interventions [27-30,43,45-47]. For example in Kilombero and Ulanga districts, Tanzania, prior to a social marketing programme only 20% of the poorest households owned a net compared to 60% for the least poor, improving to about 50% and 90% respectively three years post implementation [43,46]. However, after mass distribution alongside a measles campaign in Zambia and Ghana the difference in utilisation between the lowest and highest quintiles was much closer (78% compared to 88% in Zambia, and 62% compared to 75% in Ghana) [27,28] and in Ghana the difference was not statistically significant. Similarly, a study among rural Kenyan children after a stand-alone free distribution programme revealed no significant differences in net use across SEG (66% for poorest compared with 67% for least poor group), in contrast to pre-intervention figures of 3% and 16% in 2004 respectively when nets were predominantly available from the commercial sector [29]. More recent studies in Tanzania and Niger found that bed net and ITN possession and use significantly increased following an integrated free net distribution campaigns [43,47]. In Tanzania, bed net and ITN possession and utilisation for households with under-fives increased from 60.9% to 90.7% and from 16.5% to 37.3% respectively [43]. In Niger, ITN ownership increased from 6.3% to 65.1% following free net distribution under an integrated child health campaign [47]. These studies concluded that free net distribution via an integrated campaign is effective in rapidly and equitably increasing possession and use of bed nets. A study in rural Tanzania examined bed net distribution through markets, voucher subsidies and free nets all combined, and found these distribution strategies to be complementary [44]. In particular, the authors noted that vouchers and free bed nets rapidly increased coverage.

A free distribution campaign is now planned in Tanzania to complement the voucher scheme. Our data from rural and urban Tanzania, together with the literature from other countries described above, indicate that this may be the most effective way to increase coverage among poorer groups. Furthermore, these findings indicate the importance of targeting rural areas, and under-fives in any such programme since utilisation rates within these groups remain disturbingly low despite the fact that they suffer most from the consequences of the disease.

## Competing interests

The authors declare that they have no competing interests.

## Authors' contributions

FM: led drafting of this manuscript, supervised data collection and analysed the data. CG: helped with data analysis and co-wrote the paper. VW: conception of the DeMTAP study, helped with interpretation of results and revision of the manuscript. MW: principal investigator and commented on the manuscript draft.

## Supplementary Material

Additional file 1 **Summary statistics of PCA of household asset variables**. The data provided represent the mean, range, weight and the impact on the PCA score for each household item included in the computation of the asset-index. Click here for file
