# Supplementary material for: How equitable is bed net ownership and utilisation in Tanzania? A practical application of the principles of horizontal and vertical equity
Source: Malar J. 2009 May 21;8:109. doi: 10.1186/1475-2875-8-109 (PMC2695473; doi:10.1186/1475-2875-8-109)
Supplement: Additional file 1 — Summary statistics of PCA of household asset variables. The data provided represent the mean, range, weight and the impact on the PCA score for each household item included in the computation of the asset-index. [file 1475-2875-8-109-S1.doc]

**Additional File 1: Summary statistis of PCA of household asset variables**

| **Variable** | **Obs** | **Mean** | **SD.** | **Min** | **Max** | **Weight** | **Impact on PCA score*** |
| --- | --- | --- | --- | --- | --- | --- | --- |
| Radio | 1603 | 0.978 | 0.678 | 0 | 6 | 0.269 | 0.396 |
| Iron bed | 1603 | 2.354 | 1.632 | 0 | 10 | 0.385 | 0.236 |
| Bicycle | 1603 | 0.787 | 0.926 | 0 | 10 | 0.195 | 0.211 |
| Tin roof | 1603 | 0.205 | 0.455 | 0 | 7 | 0.312 | 0.685 |
| Television | 1603 | 0.147 | 0.383 | 0 | 3 | 0.359 | 0.938 |
| Motorbike | 1603 | 0.064 | 0.308 | 0 | 6 | 0.298 | 0.966 |
| Watch | 1603 | 1.168 | 1.338 | 0 | 10 | 0.362 | 0.271 |
| Mattress | 1603 | 1.794 | 1.778 | 0 | 13 | 0.412 | 0.232 |
| Cattle | 1603 | 0.559 | 3.475 | 0 | 70 | 0.039 | 0.011 |
| Sheep | 1603 | 0.047 | 0.442 | 0 | 7 | 0.030 | 0.068 |
| Goat | 1603 | 0.858 | 3.228 | 0 | 40 | 0.053 | 0.016 |
| Chicken | 1603 | 5.166 | 18.290 | 0 | 400 | 0.130 | 0.007 |
| Electric cooking | 1603 | 0.046 | 0.209 | 0 | 1 | 0.287 | 1.377 |
| Wood fuel† | 1603 | 0.424 | 0.494 | 0 | 1 | 0.145 | 0.294 |

***Source***: HHS Data

* This is the impact of a change from 0 to 1 for dummy variables and a unit change for continuous variables (weight/SD).

† Wood fuel refers to purchased fire wood and charcoal.

All variables are continuous variables except electric cooking and wood fuel which are binary dummy variables.
